# Polypoid Carcinoma of the Oropharynx with Stromal Ossifying Myofibroblastic Proliferation: A Case Report and Literature Review

**DOI:** 10.1155/2016/2540407

**Published:** 2016-12-05

**Authors:** Marcello Filotico, Alessandro D'Amuri

**Affiliations:** Department of Anatomic Pathology, Fondazione Card. Panico Azienda Ospedaliera, Tricase, Italy

## Abstract

A 76-year-old man reported a worsening difficulty in swallowing, leading to the inability to eat. Physical examination and CT scan revealed a polypoid mass on the posterior oropharynx and obstructing the oropharyngeal space. Histologically, the surface was ulcerated. In the underlying necrotic rim, there was active granulation tissue, and a proliferation of voluminous, globoid elements with hyperchromatic and irregular nucleus, sometimes arranged in a alveolar aggregate. The core of the lesion contained spindle-like myoid elements in interwoven bundles, with trabeculae of osteoid matrix maturing into calcified bone. Immunohistochemistry documented positivity for cytokeratins, epithelial membrane antigen, and P63 in the globoid elements beneath the necrotic rim; strong and diffuse expression of vimentin, smooth muscle actin, and CD99 and BCL2 in the spindle elements; and complete negativity for cytokeratin 5/6, high molecular weight cytokeratin (clone 34*β*E12), S100, muscle-specific actin, desmin, CD117, and anaplastic lymphoma kinase. The lesion was morphologically and immunophenotypically classified as a polypoid oropharyngeal carcinoma with ossifying myofibroblastic stromal proliferation.

## 1. Introduction

For many years, the pathologic meaning of polypoid lesions on the oropharyngeal, laryngeal, and esophageal mucosae has been debated. These lesions are characterized by a superficial ulcerated squamous carcinoma, often in situ, associated with a sarcomatoid stroma. For this reason, they are called pseudosarcomas [1]. One of us (MF) has a long interest in this type of lesion [2, 3]. The peculiar pathologic presentation of such a lesion has led us to return to the subject.

## 2. Case

**Figure 1:**
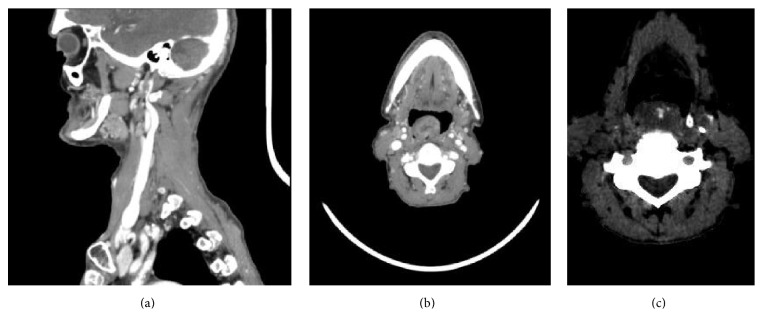
CT scans of the patient. (a) Sagittal view of head and neck. (b, c) Axial views.

A 76-year-old Italian man reported a worsening difficulty in swallowing over several months leading to the inability to eat. Physical examination revealed a polypoid mass on the posterior wall of the oropharynx. CT of the head and neck showed that the lesion had a wide base and a diameter of about 3 cm, occupying almost all the oropharynx (Figures 1(a) and 1(b)); foci of calcium deposits were also seen (Figure 1(c)).

The patient underwent surgical resection of the tumor and was then referred for chemotherapy and radiotherapy. His rapidly deteriorating conditions did not allow the full administration of the treatments, and he died five months after diagnosis.

### 2.1. Histopathological Analysis

The surgical specimen was a 2 × 1.5 × 1 cm, voluminous fleshy polypoid fragment, pink, with ulcerated surface. The material was fixed in formalin and embedded in paraffin; it was stained with hematoxylin and eosin and studied by immunohistochemistry.

**Figure 2:**
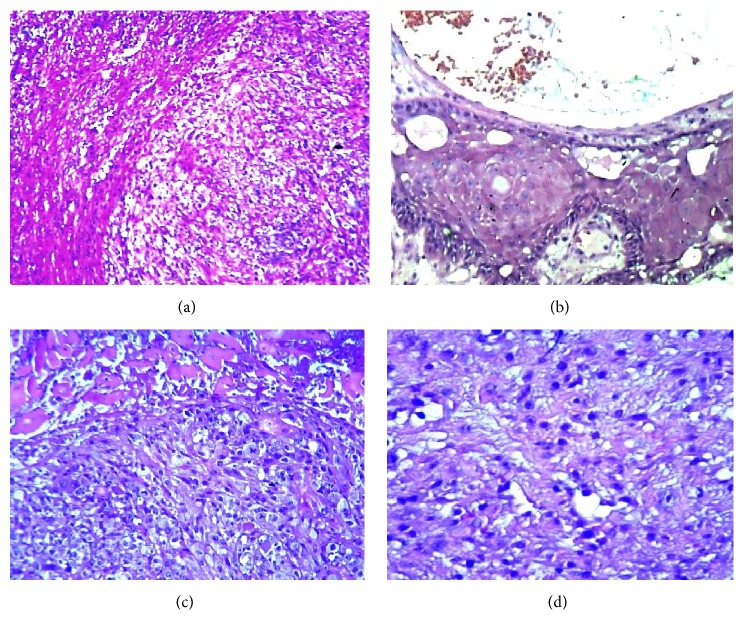
*Peripheral part of the tumor*. (a) Necrotic surface of the polypoid neoformation (40x). (b) Hyperplastic residual squamous epithelium (40x). (c) Atypical spindle and globoid elements beneath the necrotic area (100x). (d) Atypical elements in alveolar aggregation (100x).

In the specimen, we distinguished two areas: one peripheral and another deeper. The peripheral area consisted of necrotic inflammatory tissue (Figure 2(a)) on which only short sections of residual squamous surface epithelium were recognizable (Figure 2(b)). Within the necrotic outer layer, a brisk microangiogenesis by granulation tissue is present, which is intermingled groups of plump, spindled, or globoid cells, with hyperchromatic and irregular nuclei, sometimes with atypical mitosis, and aggregates in a pseudoalveolar fashion (Figures 2(c) and 2(d)).

**Figure 3:**
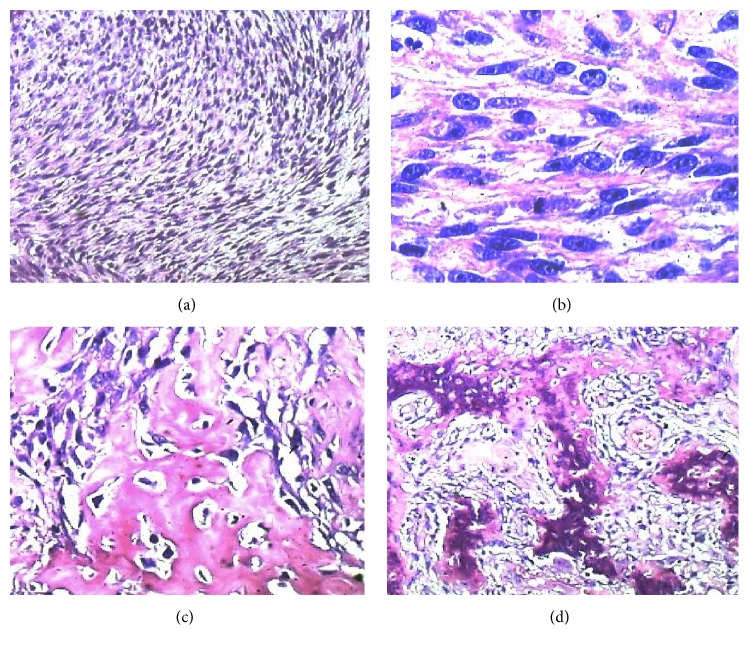
*Core of the tumor*. (a) Monomorphic proliferation of spindle elements (40x). (b) Myoid morphology (100x). (c) Osteoid trabeculae. (d) Mature trabecular bone.

The core of the lesion consisted of a proliferation of spindled, myoid-like elements, assembled in interwoven bundles, with voluminous, oval, hyperchromatic nuclei (Figures 3(a) and 3(b)). Here, the cytoplasm was abundant and amphophilic, and mitotic activity was fairly lively. In the context of the spindled proliferation, we observed trabeculae of osteoid matrix with some areas of calcified cancellous bone (Figures 3(c) and 3(d)). In the spindled core, microangiogenesis was absent.

**Figure 4:**
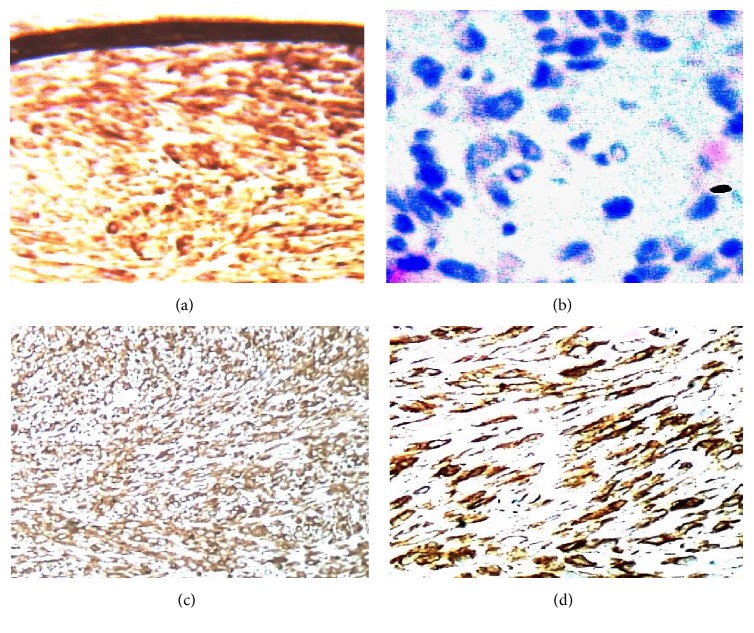
*Immunohistochemistry*. (a) Intense positivity for cytokeratin (clone AE1-AE3) of atypical elements in subepithelial site (40x). (b) Total negativity for cytokeratin (clone AE1-AE3) in the spindle elements of the core. (c) Vimentin positivity in the spindle component. (d) *α*-Smooth muscle actin positivity.

**Figure 5:**
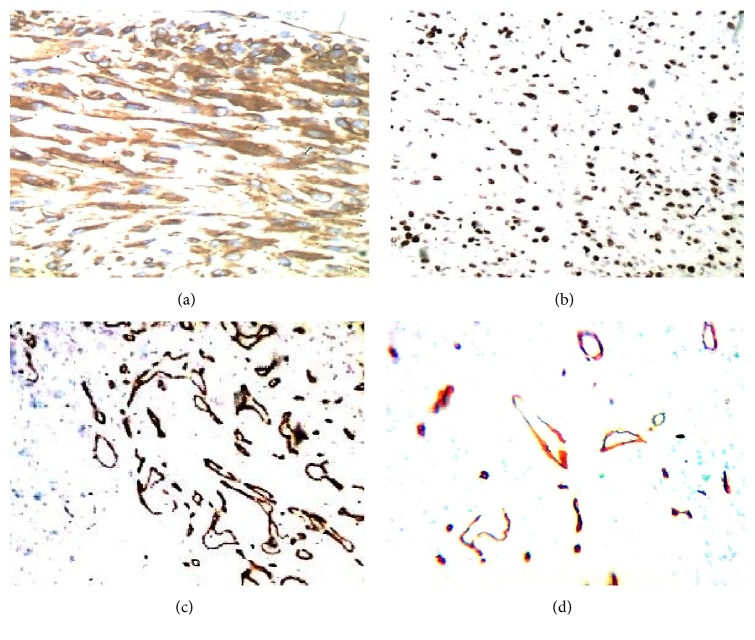
*Immunohistochemistry*. (a) Intense and diffuse positivity for CD99 in the spindle elements. (b) Ki-67 positivity in the spindle elements. (c) Positivity for CD34 in the superficial granulation tissue. (d) Positivity for CD34 in the core.

Immunohistochemical analysis (Table 1) highlighted positivity for cytokeratins (Figure 4(a)), epithelial membrane antigen, and P63, with varying intensity and frequency, in the atypical elements present in the superficial necrotic tissue, but not in the spindled core (Figure 4(b)). The spindled core expressed, with varying intensity and frequency, vimentin (Figure 4(c)), smooth muscle actin (Figure 4(d)), calponin, CD99 (Figure 5(a)), and BCL2 (focal). There was complete negativity for S100, cytokeratin 5/6, high molecular weight cytokeratin (clone 34*β*e12), muscle-specific actin, desmin, CD117, and anaplastic lymphoma kinase (ALK). Staining for Ki-67 revealed a rather high proliferation index (35%) in atypical elements expressing cytokeratins (Figure 5(b)). Staining for CD34 indicated rich microangiogenesis in the granulation tissue of the superficial necrotic band, but a paucity of vascular proliferation in the deeper area (Figures 5(c) and 5(d)).

Fluorescence in situ hybridization was used to assess the state of the* SYT* gene (18q11.2). This work indicated that the gene was not affected by translocation (data not shown).

## 3. Discussion

Since 1957, when Lane [4] first described these peculiar polypoid lesions of the upper aerodigestive tract, the debate about their meaning has not yet been exhausted, despite the remarkable progress in the fields of morphology, immunohistochemistry, and molecular biology that occurred during the nearly sixty years since the first report [5–7]. The debate essentially concerns the significance of the “sarcomatous” component that makes up the core of these lesions. Some, Lane among them, believe that the granulation tissue, under particular circumstances (carcinoma in situ, ulceration in the mucosa, and squamous epithelium), grows with abnormal characteristics, taking on sarcomatoid morphology and polypoid conformation; they call these lesions “pseudosarcomas” [3, 6, 8, 9]. Others consider the sarcomatous proliferation epithelial in nature and therefore label the lesions as “sarcomatoid carcinomas” [10–13]. Still others consider these proliferations as the sarcomatous component of a carcinosarcoma [9]. According to the literature, all three of the options are possible [8, 9, 11, 14].

The first option describes a pleomorphic cell morphology of the “sarcomatous” component, associated with an equally pleomorphic immunophenotype indicative of the histiomonocytic proliferation and the microangiogenesis of the granulation tissue (i.e., positivity for vimentin, actin, calponin, desmin, CD31, CD34, and CD68), without expression of epithelial antigens. The second, based on the “sarcomatous” component's expression of cytokeratins (particularly high molecular weight cytokeratin) which is often associated with the expression of mesenchymal antigens like vimentin, focuses on the carcinomatous nature of the proliferation—sarcomatoid carcinoma. The third, in addition to considering the carcinomatous proliferation, emphasizes the unmistakable characteristics of a mesenchymal malignancy with sometimes heterologous characteristics (resembling rhabdo-, leiomyo-, and chondroosteosarcomas) with a conforming immunophenotypic profile.

The particular features in this case are (1) polypoid conformation, (2) ulceration, (3) evidence of an epithelial neoplasm in the context of necrotic tissue, well highlighted by the immunohistochemical analysis, (4) the core, consisting of a monomorphic proliferation of spindled, myoid-like elements, between which there was focal deposition of osteoid material and mature cancellous bone, and (5) strong positivity of the spindled component for vimentin, *α*-smooth muscle actin, CD99, and BCL2.

This case is not a typical Lane's pseudosarcoma, insofar as the sarcomatoid component lacks morphologic and immunophenotypic features characteristic of granulation tissue, which are present, on the other hand, in the peripheral necrotic tissue. This case does not fulfill the immunophenotypic characteristics of a sarcomatoid carcinoma because the sarcomatoid component does not express epithelial antigens. The possibility of a monophasic synovial sarcoma of the oropharynx [15], considering the positivity for cytokeratins, CD99, and BCL2, or of its variant ossificans form [16, 17], is discarded by the absence of an* SYT* translocation. The presence of contractile filaments, throughout most of the bulk of the tumor, provides evidence for the myofibroblastic nature of the proliferation. The bone component is the result of the maturation of an osteoid matrix which originated between the spindle cells (Figure 3(c)) leading to the formation of mature trabecular bone (Figure 3(d)). This type of direct ossification without cartilaginous precursors is reminiscent of myositis ossificans, in which the cellular component is represented by myofibroblasts.

In summary, the tumor in question has only some of the characteristics of the most common polypoid malignancies of the upper aerodigestive tract, variously labeled as pseudosarcomas, sarcomatoid carcinomas, and carcinosarcomas. A carcinomatous component was demonstrated morphologically and immunophenotypically, in the superficial necrotic tissue (Figures 2(c), 2(d), and 4(a)). The core of the lesion does not have the characteristics of atypical granulation tissue or those of a sarcomatoid carcinoma, but rather that of a monomorphic proliferation of myofibroblasts.

This morphologic and immunophenotypic profile may be compared to that of a group of lesions that goes under the umbrella definition of “pseudosarcomatous myofibroblastic proliferations” (PMPs) [18–22]. PMPs are similar to nodular fasciitis in its various expressions (proliferative fasciitis, proliferative myositis, and ossificans) [19, 21] and from time to time are labeled under various designations such as visceral fasciitis, pseudosarcomatous fibromyxoid tumor [19], spindle cell pseudomalignant proliferation, postoperative spindle cell nodule [23], and inflammatory pseudotumor [18]. These lesions most often occur in the genitourinary system (bladder, prostate, ureter, vagina, and vulva) [24–26] but can occasionally arise in the gastrointestinal tract [27, 28] or in the organs of the upper aerodigestive tract (pharynx, larynx, nasal cavities, and mouth) [29, 30]. They should be clearly distinguished from the inflammatory myofibroblastic tumor (IMT) [31, 32], and for this reason the confusing adjective inflammatory should be avoided in their description.

Unlike nodular fasciitis, where there are clonal aberrations in different areas of the genome [33–35], in PMP these have not been reported. In 50% of the cases, the lesions are positive to ALK, which usually does not correspond to any ALK rearrangements detectable with FISH. When this occurs, these cases are classified as IMT [21].

The positivity for CD99 and BCL2 in this case suggests that the tumor is quite different from classic myofibroblastic proliferations, such as nodular fasciitis, fibromatosis, and dermatofibroma, as well as from benign and malignant smooth muscle proliferations, which are all uniformly negative for these antigens. However, with current knowledge, we are unable to define the lesion, just as we are unable to determine whether the proliferation is neoplastic or merely reactive.

The analysis of this case shows that the “sarcomatoid” proliferation associated with the polypoid carcinoma of the high aerodigestive tract, in addition to a reactive (Lane's tumor) or neoplastic epithelial (sarcomatoid carcinoma) process, may be due to other proliferative conditions, which in this case has the morphological and immunophenotypic characteristics of a myofibroblastic proliferation. To date, lesions of this type associated with a polypoid carcinoma of the upper aerodigestive tract have not been reported in the literature.

## Figures and Tables

**Table 1:** Immunoreactivity of the neoplasm.

Antigen (antibody clone)	Antibody^a^ dilution	Immunoreactivity	
		Superficial layer	Core
ALK	1 : 25	−	−
BCL2	1 : 50	−	+f
Calponin	1 : 50	−	±
CD31	1 : 20	+gt	−
CD34	1 : 20	+gt	−
CD99	**1 : 250**	−	+
CD117	1 : 400^b^	−	−
CK (clone AE1/AE3)	1 : 50	+f	−
CK 5/6	1 : 50	−	−
CKhmw (clone 34*β*E12)	1 : 100	−	−
Desmin	1 : 50	−	−
EMA	1 : 50	±	
Muscle-specific actin	1 : 50	−	+
Ki-67	1 : 75	35% positive	25%
P63	1 : 75	±f	
S100	1 : 000^b^	−	−
Smooth muscle actin	1 : 50	−	+
Vimentin	1 : 50	−	+

^a^All antibodies are from Dako and monoclonals unless otherwise indicated. ^b^Polyclonal antibody.

CK, cytokeratin; +, positive; −, negative; ±, positivity observed in <15% of cells in the sample; gt, granulation tissue; f, focal.
